# Plasticity Within the Obligatory Folding Nucleus of an Immunoglobulin-like Domain

**DOI:** 10.1016/j.jmb.2007.09.088

**Published:** 2008-01-11

**Authors:** Ilkka Lappalainen, Michael G. Hurley, Jane Clarke

**Affiliations:** University of Cambridge Department of Chemistry, MRC Centre for Protein Engineering, Lensfield Rd, Cambridge CB2 1EW, UK

**Keywords:** Ig, immunoglobulin, fnIII, fibronectin type III, CAfn2, the second fnIII domain of chitin A1 from *Bacillus circulans*, TNfn3, the third fnIII domain of human fibronectin, folding nucleus, protein folding, phi-value analysis, Ig domain

## Abstract

A number of β-sandwich immunoglobulin-like domains have been shown to fold using a set of structurally equivalent residues that form a folding nucleus deep within the core of the protein. Formation of this nucleus is sufficient to establish the complex Greek key topology of the native state. These nucleating residues are highly conserved within the immunoglobulin superfamily, but are less well conserved in the fibronectin type III (fnIII) superfamily, where the requirement is simply to have four interacting hydrophobic residues. However, there are rare examples where this nucleation pattern is absent. In this study, we have investigated the folding of a novel member of the fnIII superfamily whose nucleus appears to lack one of the four buried hydrophobic residues. We show that the folding mechanism is unaltered, but the folding nucleus has moved within the hydrophobic core.

## Introduction

Studies of structurally related proteins have clearly indicated that for many proteins the folding mechanism is determined primarily by the native state topology.^1,2^ This is evident from comparative Φ-value analyses of proteins that share similar folds but are very different in sequence. Such studies include representatives from all protein classes; all-α proteins,^3–9^ all-β proteins,^10–22^ and mixed α/β proteins.^23–28^ The folding mechanisms of these proteins range from purely hierarchical, where secondary structural elements form before any tertiary structure, to pure nucleation-condensation, where secondary and tertiary structure form concomitantly.^29^ A study of representative members of the homeodomain superfamily family has suggested that their folding mechanisms are dependent on inherent secondary structural propensity, and the authors propose that all folding mechanisms are in fact variations of the same theme:^3^ as the propensity for forming secondary structures decreases, the folding mechanism shifts from pure hierarchical to polarized transition states, and ultimately to the classical nucleation-condensation mechanism first shown for CI2.^30^ Separate investigations have suggested that a folding nucleus consists of obligatory and critical components: the specific interactions necessary to establish the correct topology form first, followed by a subset of surrounding residues that provide the critical stabilising interactions.^31–33^

As part of the “fold approach” we have studied the folding of a number of proteins with an Ig-like fold,^16–18,34–36^ all of which are composed of two anti-parallel β-sheets packed against each other. The deep hydrophobic core is always formed from the packing of the four central B, C, E and F β-strands,^37^ but the number and position of the edge strands varies between the superfamilies.^38^ Although proteins in different superfamilies share the same fold, they are apparently unrelated in sequence and are found in proteins with a wide variety of functions. The stabilities of the Ig-like proteins studied to date range from about 1 kcal mol^− 1^ to 9 kcal mol^− 1^, and the folding rate constants vary by six orders of magnitude. However, there is a correlation between folding rate and thermodynamic stability, which suggests that the interactions that are critical in stabilising the fold also govern the folding process.^34^

**Figure 1:**
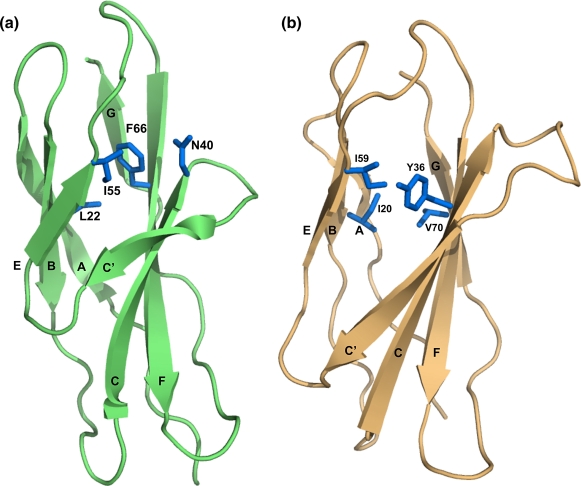
The structure of the CAfn2 (left, green, PDB code 1K85) and TNfn3 (right, orange, PDB core 1TEN). The side-chains forming the putative folding nucleus in both structures are shown in blue. In most fnIII domains these residues form a ring of interactions deep within the core, as is shown for TNfn3. The polar side-chain of N40 in CAfn2 is not interacting with the other residues in the folding nucleus. The CAfn2 has the same topology as all other fnIII domains: seven β-strands that arrange into two β-sheets.^55–57^ The first sheet is formed of the A, B and E-strands, and the second sheet is formed of the C′, C, F and G-strands. Figures 1, 5 and6 were made using PyMol [http://pymol.sourceforge.net/].

All members of the fold studied to date fold *via* a nucleation-condensation mechanism where the obligatory folding nucleus comprises a set of structurally equivalent buried hydrophobic residues in the B, C, E and F-strands that form a “ring” of interactions in the core (Figure 1). Early packing of the residues, which are distant in sequence, ensures formation of the correct native state topology.^35,36^ The critical nucleus surrounds this obligatory nucleus, but the degree of structure formation varies between different proteins.

The residues that form the obligatory folding nucleus are highly conserved within immunoglobulin domains but are conserved only in terms of residue type in fibronectin type III (fnIII) domains. However, there are rare examples of proteins that appear to have a disparate nucleation pattern. Here, we have identified a fnIII domain in which one of the hydrophobic residues in the conserved folding nucleus has been replaced by a surface polar residue, and we ask how the folding mechanism has been affected. An extensive protein engineering Φ-value analysis reveals that the folding mechanism is unaltered, but that a spatially different set of core residues is used to form the obligatory folding nucleus, where interactions within each sheet establish the correct hydrogen bond registry between the core β-strands. Subsequent interactions between two such pairs are able to bring the β-sheets together and set up the complex Greek key topology.

## Results

### Residue conservation within the folding nucleus of fnIII domains

A non-redundant multiple sequence alignment from Pfam was used to analyse the residue conservation in the putative folding nucleus of fnIII domains.^39^ These nucleation positions were identified through comparison with the third fnIII domain from human tenascin (TNfn3), which has been studied extensively in our laboratory. In most fnIII domains (73%), all four residues in the proposed folding nucleus positions are hydrophobic. Further analysis reveals that where there is a polar residue in one of these folding positions, it is almost invariably in the C or E strand, and these hydrophilic residues are usually arginine or lysine. These can act as hydrophobic residues, since the long aliphatic side-chains can traverse the core and allow the charged terminus to reside on the surface of the protein.^40^ Small hydrophilic residues, such as asparagine or aspartate, are found very rarely (only in ∼ 3% of all cases).

**Figure 2:**
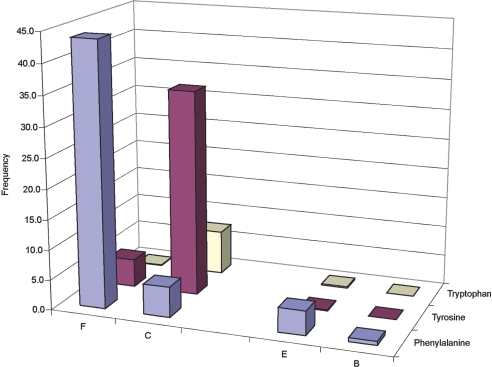
The fnIII sequences containing only a single aromatic residue within the predicted obligatory folding nucleus. The frequency of the appearance of a given amino acid in any position is shown on the *y*-axis. The majority of the sequences have either phenylalanine at the F-strand or tyrosine at the C-strand folding position.

Most fnIII domains (65%) have a single aromatic residue in the proposed folding nucleus. Analysis of the distribution of aromatic residues in the four obligatory folding nucleus positions shows clearly that aromatic amino acids are located preferably within the C–F sheet (Figure 2). Furthermore, the type of aromatic residue present is affected by the solvent accessibility of the β-strand: the C-strand position is partly solvent-accessible, and hence the majority of aromatic residues occurring within this strand are tyrosine (thereby allowing hydrogen bonding of the hydroxyl group with solvent molecules). In contrast, the F-strand position is deep within the core and phenylalanine is almost always the aromatic residue of choice. Approximately 20% of the sequences have more than one aromatic residue in the folding nucleus, and again these residues are almost exclusively located in the C–F sheet (86% of all such sequences). This asymmetry is likely caused by the presence of an adjacent conserved tryptophan in the B-strand that is essential for stability but not involved in the folding nucleus.^16,18^ Interestingly, about 15% of the fnIII domains appear to fold without any aromatic residues at the supposed folding positions, suggesting that a large side-chain is not crucial for the formation of the obligatory nucleus.

### Selection of CAfn2 as a candidate

All known fnIII structures were surveyed to find a candidate protein that was missing a hydrophobic residue at one of the four putative folding positions. Only one candidate protein was identified, the second fnIII domain in chitin A1 from *Bacillus circulans* (CAfn2), which has a surface-exposed asparagine in the putative nucleus position in the C-strand. It has an aromatic residue in the F-strand folding position (Phe66).^41^

**Figure 3:**
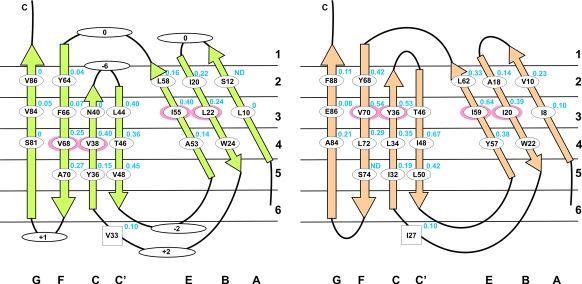
A simplified representation of the CAfn2 (left, green) and TNfn3 (right, orange) structures showing the mutated positions within each strand. The core of the proteins is divided into six layers, residues within the same layer pack against each other on opposite β-sheets. The differences in loop lengths between CAfn2 and TNfn3 are shown for each loop in the CAfn2 representation (+ means the CAfn2 loop is longer, –– means the CAfn2 loop is shorter). The Φ-values are marked in blue for each position. The obligatory folding positions are shown as a red oval.

In this work we intended to compare the folding of CAfn2 with TNfn3, as this is the most extensively studied “typical” fnIII domain. The structure of CAfn2 was superimposed on the structure of TNfn3 to reveal a RMSD of only 1.7 Å over all structurally equivalent positions (69 residues), even though the two proteins have just 13% sequence identity (Supplementary Data Figure 1). The major differences are restricted to the loops and turns, which are different in length in the two proteins. CAfn2 has the same Greek key topology as all other fnIII domains, (Figure 1), and possesses both the highly conserved Trp residue in the B-strand and the conserved tyrosine corner motif (Supplementary Figure 1).^42^ A comparison of the structures of CAfn2 and TNfn3 reveals that the lengths of the highly conserved EF-loop and the AB-turn are identical in these proteins, whereas the rest of the loops show some variation. Most notably the C and C′-strands are shorter in CAfn2 and are joined by a short tight turn (Figures 1 and 3). The packing interactions are almost identical in the two proteins, with the majority of the contacts being made within the same sheet, and the interactions between sheets occurring in “layers”.^18^ To help visualize the interactions that occur within the proteins, all residue positions are described according to both the β-strand and the core layer in which they reside (Figure 3).

Importantly, however, inspection of the supposed obligatory folding nucleus of CAfn2 clearly shows that the C-strand residue, N40, does not pack against the other putative nucleus residues (Figure 1). How, therefore, does this domain fold?

### Characterization of wild-type CAfn2

The equilibrium stabilities of wild-type CAfn2 and all its mutant proteins were determined through the use of standard denaturation curves fit to a two-state equation.^43^ From a number of repeated measurements of wild-type CAfn2 the free energy of unfolding (Δ*G*_D–N_) was estimated to be 6.7( ± 0.3) kcal mol^− 1^ at pH 5.0 and 25 °C.

**Figure 4:**
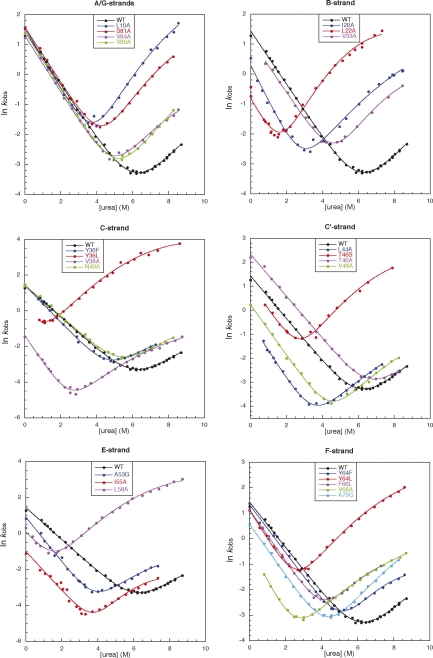
Chevron plots for each mutation according to the β-strand. The observed rate constants (*k*) are measured in s^−^ ^1^ and the concentration of urea ([urea]) is measured in M.

Kinetic studies reveal a single unfolding phase, but two refolding phases. Since the slower phase accounts for less than 15% of the total amplitude, and is apparently independent of the concentration of denaturant, it was attributed to proline isomerisation. Both arms of the chevron plot are linear (Figure 4), and the β_T_ for wild-type CAfn2 is estimated to be 0.56. This is similar to that of TNfn3,^44^ indicating that the two transition state structures are of a similar compactness.

### Effect of mutations

Using the Φ-value analysis of TNfn3 as a basis, a total of 23 non-disruptive mutations were made throughout the CAfn2 protein. Where possible, all mutations were conservative deletions.^45^ Contacts lost upon mutation are given in Table 1. The difference in free energy between wild-type and mutant (ΔΔ*G*_D–N_) was calculated using an average *m*-value, <*m*>, of 1.15(± 0.02) kcal mol^− 1^ M^− 1^. It has been shown that *m-*values are hard to determine with accuracy (the range of *m*-values observed is typical for that observed in most large-scale protein engineering studies) so that use of an average *m*-value reduces the error in ΔΔ*G*.^46^ Most mutations are destabilizing but ΔΔ*G*_D–N_ values range between − 1.5 kcal mol^− 1^ and + 6.0 kcal mol^− 1^.

Chevron plots for all mutants are shown in Figure 4, with the kinetic data given in Table 2. Using these data, Φ-values were calculated from refolding data at 0 M denaturant. Note that for three highly destabilized proteins (L22A, Y36L and L58A) there were too few data points in the refolding arm to determine the gradient (*m*_kf_) accurately. In these cases an average *m*_kf_ (1.06 M^− 1^) was used to fit the data. Note that this has no effect on the final Φ-value determined at 0 M denaturant. Only one mutant, V38A, has a folding *m-*value that is significantly different from this mean value.

Several mutant proteins exhibit roll-over in the unfolding arm, and some mutants have increased *m*_ku_ values. Such behavior in unfolding has several possible explanations. It has been ascribed to “Hammond” behaviour, where there is a broad transition state barrier,^47^ or to population of a high-energy intermediate.^48^ We do not have sufficient data to distinguish these two possibilities and, since there is no roll-over in the wild-type protein, it is not possible to determine Φ-values for the “late” transition state using unfolding data. However, the model used has no effect on analysis of the “early” transition state, at 0 M denaturant.^49^

### Structure of the transition state

A number of positions in each β-strand of CAfn2 were probed using Φ-value analysis. Φ is a measure of the extent of structure at a given residue in the transition state (‡). A Φ-value of 1 indicates that the interactions are fully formed in ‡, whereas a Φ-value of 0 indicates that the structure is as unfolded in ‡ as in the denatured state. The precise interpretation of fractional Φ-values is ambiguous but is usually taken to mean that the residue is partly structured in ‡.^50^ However, it is generally accepted that, particularly when comparing homologous proteins, the best approach is to look at patterns of Φ-values rather than considering the absolute values of individual residues.^2^

**Figure 5:**
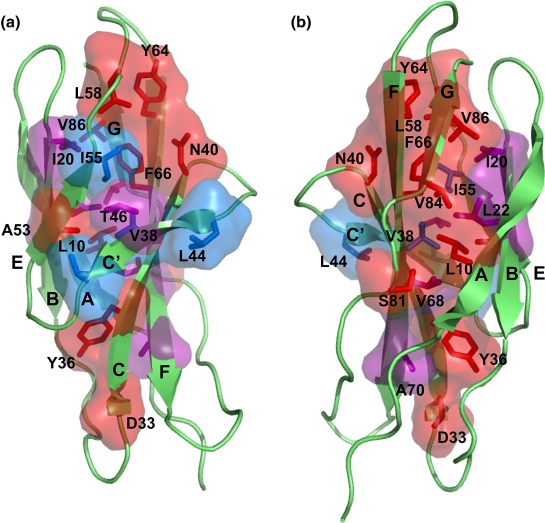
The CAfn2 structure showing the ϕ-values (high, blue, Φ ≥ 0.4; medium, magenta, 0.2 < Φ < 0.4; and low, red, Φ ≤ 0.2). (a) The front view of CAfn2 (the CAfn2 structure is oriented as in Figure 1). (b) The rear view of CAfn2.

The CAfn2 Φ-values range from 0 to 0.5, indicating that none of the positions analyzed is completely structured at the transition state (Table 2). In general, the Φ-values in the A and G-strands are close to 0, while those in the central B, C, C′, E and F β-strands are higher, and the Φ-values in these central strands are higher in the central layers of the core than at the extremes (Figure 3), as observed in TNfn3,^18^ the tenth fnIII domain of fibronectin (FNfn10)^16^ and the titin immunoglobulin domain TI I27.^17^ The Φ-values were classified into low (Φ ≤ 0.2), medium (0.2 < Φ < 0.4) and high (Φ ≥ 0.4) classes. These Φ-values are mapped onto the CAfn2 structure in Figure 5.

#### A and G-strands

All mutations in the A and G-strands gave low Φ-values, indicating very little structure formation in these strands in the transition state (Table 2; Figures 3 and 4). Both mutated sites in the A-strand, L10A(A3) and S12A(A2), pack onto the neighbouring B-strand, whilst inter-sheet interactions are formed with residues from the F and G-strands (Table 1). The ΔΔ*G*_D–N_ for the S12A(A2) mutation is too low for a reliable Φ-value to be determined. The three residues probed in the G-strand, S81(G4), V84(G3) and V86(G2), make interactions mainly with residues from the A and F-strands (Table 1).

#### Strands B, C, E and F

All core positions in CAfn2 were mutated, with the exception of W24 in the B-strand. The distribution of Φ-values reveals that the hydrophobic core is only partially formed in the transition state (Table 2; Figures 3 and 4). The highest values occur at positions V38(C4) and I55(E3), with moderate Φ-values at four other positions: I20(B2), L22(B3), V68(F4) and A70(F5).

#### C′-strand

The C′-strand is connected by two short loops to the central C and E-strands. Three of the four mutations within this strand, L44A(C′3), T46A(C′4), and V48A(C′5), have high Φ-values (Table 2; Figures 3 and 4). T46 and V48 interact mainly with the buried residues from the C and E-strands, whereas the L44 contacts are limited to residues within the C and C′-strands (Table 1).

## Discussion

### CAfn2 folds by a nucleation-condensation mechanism

There has been much discussion of folding mechanisms in recent years. The two “extremes” are represented by the framework model, where local secondary structure forms before tertiary structure, and nucleation-condensation, where secondary structure and tertiary structure form concomitantly. Such extremes have distinct patterns of Φ-values. In the framework model, Φ-values will fall into two groups, one set close to 1 and the other close to 0. This has been termed a polarised transition state. In a nucleation-condensation mechanism, the transition state structure will be more diffuse, involving most of the protein, and Φ-values will all be generally between 1 and zero. Furthermore, in the nucleation condensation mechanism, the pattern of Φ-values is generally distinctive, with a subset of residues having slightly higher Φ-values, with Φ-values gradually becoming lower as structure condenses around the early “nucleus”. The pattern of Φ-values shows CAfn2 to have a diffuse nucleus, with two-thirds of the residues having Φ-values between 0.10 and 0.45. Moreover, these are arranged in the structure as one would predict from a nucleation-condensation pattern, with higher Φ-values at the centre of the core, becoming lower towards the edges of the molecule. This suggests strongly that CAfn2 folds, as do other Ig-like proteins, *via* a nucleation-condensation folding mechanism. However, again like other Ig-like proteins, there is a significant number of residues, in the peripheral A and G-strands, and in loops that have Φ-values close to 0, suggesting that in the final stage of folding these peripheral strands and loops pack onto the central region of the protein.

### Identification of the obligate (embryonic) folding nucleus

Oliveberg and co-workers have suggested that residues that constitute the transition state for folding in a nucleation-condensation mechanism might be divided into two sets.^31,32^ The first set of residues make up the “embryonic” or obligate folding nucleus, defined as the set of primary contacts that are obliged to form to establish the topology of the protein. The second set is the residues that pack onto this embryonic nucleus forming the “critical contact layer”, providing sufficient interactions to drive the folding process downhill. Note that residues that form the embryonic nucleus have to form a network of contacts that establish the topology of the protein, but that residues in the critical contact layer may contribute significantly towards stabilising the transition state for folding. Identifying the most likely obligate folding nucleus from a pattern of Φ-values is non-trivial, especially for complex Greek key structures. This has been discussed in detail.^18^ In summary, it is not possible simply to “pick” residues with the highest Φ-values as being those that form the folding nucleus: one also has to consider the packing of the residues. In TNfn3, as is observed here for CAfn2, residues in the B-strand have generally low Φ-values compared to the Φ-values in the other central β-strands. This does not necessarily mean that the residues in the B-strand are less important for folding. Residue L22(B3) in CAfn2, for example, forms about half of its inter-strand contacts with residues in the A and G-strands, which have Φ-values of ∼ 0. Thus, the contacts with the C (V38), E (A53 and I55) and F (F66 and V68) strands must be more formed than the moderate Φ-value would indicate. Similarly, the high Φ-values in the C′-strand probably reflect the fact that the residues in this strand make the vast majority of their tertiary contacts with residues in the C and E-strands, which are themselves partially formed. In TNfn3 it was suggested that this C′-strand is “obliged” to fold when the adjoining C and E-strands pack together. Thus, we would now, following the nomenclature suggested by the Oliveberg model, assign residues in the C′-strand to the critical contact layer and not to the obligatory embryonic nucleus.

For TNfn3, the residues with the highest Φ-value in the B, C, E and F-strands were initially chosen as putative nucleus residues. Examination of the structure showed that these residues are all found in the same core layer, and that they pack to form a “ring” of interactions in the core of the protein. It was suggested that this “obligatory” nucleus alone was sufficient to establish the topology of the native protein. This picture of the folding transition state was confirmed by subsequent restrained molecular dynamics simulations.^36^ A similar method has been used to identify the folding nucleus in a structurally related immunoglobulin domain.^17,35^

**Figure 6:**
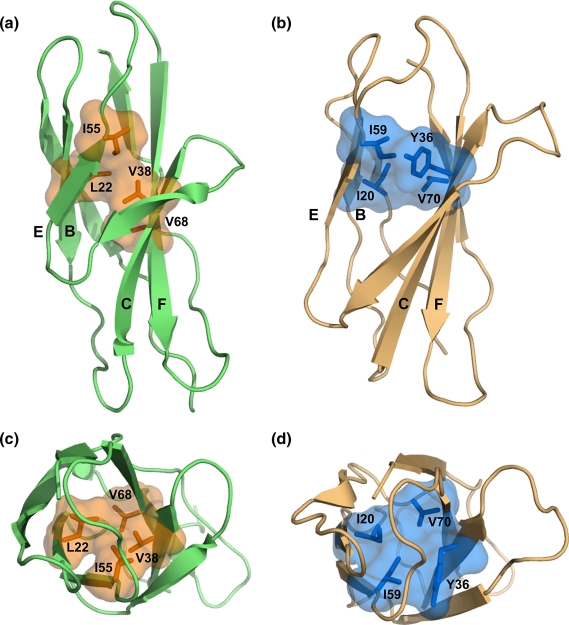
(a) and (c) The obligatory folding nucleus of CAfn2 has moved within the hydrophobic core in comparison to (b) and (d) the structurally conserved positions for TNfn3. The molecules are oriented as in Figure 1. The novel obligatory folding nucleus of CAfn2 is based on the Φ-values and contact maps between the residues in the hydrophobic nucleus.

Using the same strategy, the Φ-value pattern of CAfn2 was investigated to identify the putative obligate folding nucleus, a set of residues with significant Φ-values that interact such that these interactions are sufficient to establish the topology of the protein. The layer that contains residues with consistently high Φ-values in the B and E-strands is layer 3 (I55 and L22), as in TNfn3. However, for the C and F-strands, the layer with the highest Φ-values is layer 4 (V38 and V68). Examination of the structure of CAfn2 shows that although residues L22 and I55, and V38 and V68 sit in different core layers, these four residues are still able to pack together in the centre of the core to form a ring of contacts (Figure 6). We suggest that the residues surrounding the obligate nucleus, residues in the C′-strand and more peripheral residues in the B, C E and F-strands, pack onto these obligate nucleus residues and, together, form the critical contact layer required to stabilise the transition state structure sufficiently to drive folding.

There is a caveat we should make. We note that in CAfn2 it is more difficult to select which residues are likely to form part of the obligate nucleus than it was in TNfn3. Consider the B-strand. I20(B2) exhibits a Φ-value that is slightly lower, but is within error of L22(B3). However, I20 forms no contacts with the nucleating residues from the opposite sheet, (V38(C4) and V68(E4)), suggesting that it does not form part of the obligatory nucleus that establishes the topology of the molecule. Furthermore, as was the case in TNfn3, we were unable to determine a Φ-value for the highly conserved Trp in the position B4. Simulations confirmed for TNfn3 that this Trp residue had a low Φ-value (as we had inferred from the pattern of Φ-values surrounding the Trp residue). Trp 24 makes 150 side-chain–side-chain contacts in CAfn2, and 65% of these contacts are with residues that have Φ-values that are (or are predicted to be) low (Φ ∼ 0.15, 52 contacts) or zero (46 contacts). Less than one-third of the contacts made by Trp24 are with residues in the putative obligatory nucleus (with L22, V38 and V68) and no contact is made with I55. Thus, we tentatively propose that if Trp24 does have a role in the folding nucleus, it is more likely to be in the critical layer than in the topology-defining obligate nucleus. Also consider residue A70 in the F-strand in position F5. The Φ-value for this residue is very slightly higher than that for V68 in layer F4. However, Ala to Gly mutation must be considered to be non-conservative and, furthermore, Ala70 makes no contact within the proposed obligate nucleus i.e. it cannot have a role in establishing the Greek key topology.

### Comparison of the transition states of CAfn2 and TNfn3

**Figure 7:**
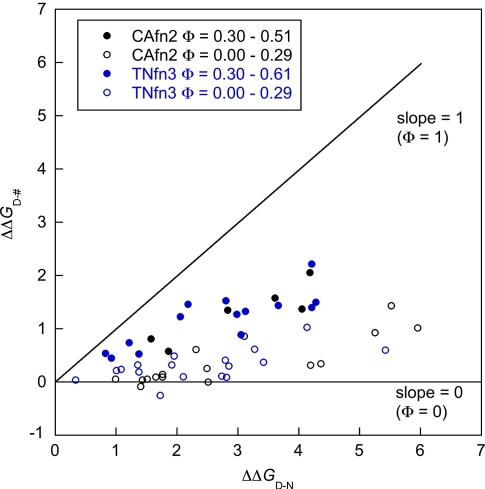
Brønsted analysis of TNfn3 and CAfn2 mutants, showing the plot of ΔΔ*G*_D–‡_*versus* ΔΔ*G*_D–N_. The separation of data points into two discrete populations shows that the folding nucleus of both proteins is not a uniformly expanded form of the native state. The central core of the protein forms early and the peripheral regions pack after the transition state for folding.

The Φ-values for TNfn3 are generally higher than those in CAfn2, ranging from 0 to 0.6;^18^ however, the pattern of Φ-values is similar. For both proteins, the mutational results can be separated into two classes. The first group consists of residues in the central β-strands, which show significant formation of structure in the transition state. The second group consists of mutations probing the terminal A and G-strands, and residues from the extremities of the central strands. These two populations are clearly observed in a Brønsted plot (Figure 7).

**Figure 8:**
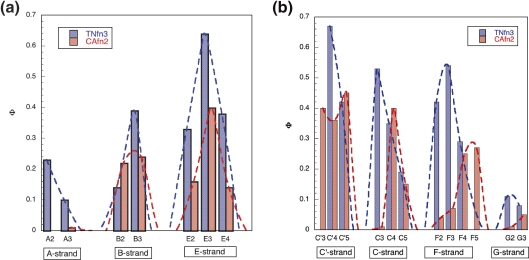
Comparison of the Φ-value patterns in CAfn2 (red) and TNfn3 (blue). (a) The pattern of Φ-values in the A–B–E strand is the same with the highest Φ-values falling in layer 3. (b) In the C′-C-F-G sheet, however, the residues with the highest Φ-values in CAfn2 are in a lower layer than in TNfn3.

Nevertheless, there are important differences between the two domains, which are apparent in the pattern of Φ-values (Figure 8). In the C–F-sheet, the highest Φ-values in TNfn3 are found in core layer 3 (V70, 0.54; Y36, 0.53), with the Φ-values in layer 4 being significantly lower (L72, 0.29; L34, 0.35). However, in CAfn2 the Φ-values in layer 3 are very low (F66, 0.07; N40, 0.01), whereas the Φ-values in layer 4 (V68, 0.25; V38, 0.40) are significantly higher (Figures 3 and 8). This indicates that the absence of a buried hydrophobic residue in position C3 has forced the obligate folding nucleus of CAfn2 to “migrate down” one layer within the core (Figure 6). Perhaps unexpectedly, a corresponding “downwards migration” has not occurred in the B–E-sheet; (even if Trp24 was important, the Φ-value for A53(E4) is unambiguously low (0.14)). Such migration is not necessary; analysis of the CAfn2 structure shows clearly that residues L22(B3) and I55(E3) form significant interactions with V68(F4) and V38(C4) in the opposite sheet. Thus, these inter-sheet interactions would be sufficient to establish the Greek key topology.

Further support for this migration hypothesis comes from Φ-values in the EF-loop. TNfn3 exhibits moderate Φ-values in this loop, (Y68(F2), 0.42; L62(E2), 0.33), which indicates that it is significantly structured in the transition state. It was argued that this loop is “obliged” to be structured in the transition state of TNfn3 to allow for formation of the adjacent folding nucleus: the more distant BC-loop exhibits lower values. However, in CAfn2 the folding nucleus has shifted away from the EF-loop (Figure 6) and consequently it is less restrained within the transition state (Φ-values for Y64(F2) and L58(E2) are 0.04 and 0.16, respectively).

Both TNfn3 and CAfn2 display high Φ-values in the C′-strand. We suggest that these residues are not involved in the obligatory folding nucleus, but result from short CE-loops that force the C′-strand to pack as the nucleus forms;^18^ thus, these residues form part of the critical contact layer. In CD2d1, an immunoglobulin Ig variable domain, the nucleating C and E-strands are joined by a much longer loop comprising three β-strands, C′, Cʺ and D. In this case these strands do not pack until late in folding.^18,19^

In summary, for both proteins we observe the formation of a specific nucleus in the core of the protein involving formation of long-range tertiary contacts between a single residue from each of the B, C, E and F-strands. Formation of this “obligate” nucleus establishes the topology of the protein. Other residues pack around this obligate nucleus to form the critical contact layer until sufficient contacts have formed to surmount the free-energy barrier. This is typical of a nucleation-condensation folding mechanism. The peripheral strands and the loops pack late, mainly after the rate-limiting step for folding.

### Conclusion: plasticity within the obligatory folding nucleus in Ig-like domains

Unlike other classes of proteins, such as the homeodomain proteins, all Ig-like proteins appear to fold by the same, nucleation condensation mechanism. The obligate nucleus is defined by the interactions that are necessary to establish the complex Greek key β-sheet topology of the native state. Previous biophysical studies of members of the Ig-like fold have shown that this folding nucleus always comprises a ring of interacting residues within the hydrophobic core: one residue from each of the B, C, E and F-strands. Whereas the obligatory nucleus in the immunoglobulin superfamily proteins is highly conserved and is based around the invariant tryptophan located within the C-strand, members of the fnIII superfamily show more variability. Instead of restricting a particular structural position to a specific amino acid, each position simply needs to possess a hydrophobic residue. Here, we have shown that the fnIII nucleus is more flexible still, and that when this pattern of residue conservation is lost upon mutation, fnIII proteins can “migrate” the folding nucleus, thereby revealing plasticity in the early stages of the folding process, while retaining the same folding mechanism.

Such plasticity in the folding of Ig-like proteins has been observed previously; the Ig domain TI I27 has been shown to fold by alternative, parallel pathways.^51^ Although the wild-type protein folds only through one pathway under physiological conditions, extremes of temperature and denaturant, or mutations within its obligate folding nucleus also result in a switch of folding pathway. Lindberg and Oliveberg have suggested recently that a “malleable” protein folding energy landscape will allow proteins to retain efficient folding during the course of evolution, even though the finer details of the folding pathway are dependent on individual sequence.^52^ It is possible that the ability of these Ig-like domains to alter their folding pathway on mutation has contributed to the success of this fold, and has contributed to its abundance in the proteome (over 40,000 Ig-like domains are listed in the current Pfam database).

## Materials and Methods

### Protein expression and purification

The fibronectin type III domain used in this work consists of 88 residues (SwissProt P20533, residues 559–646, PDB 1K85) of the *Bacillus circulans* chitinase A1. The synthetic gene was produced using overlapping primers and standard PCR techniques, and was inserted into a modified version of pRSETA vector (Invitrogen) containing an N-terminal His-tag followed by a thrombin cleavage site. Site-directed mutagenesis was performed using the QuikChange Kit (Stratagene). The identity of wild-type and mutants was confirmed by DNA sequencing.

Protein expression was carried out in *Escherichia coli* C41 cells.^53^ Transformed cells were grown to an absorbance at 600 nm of 0.6 at 37 °C before induction with IPTG and growth overnight at 28 °C. The cells were harvested and lysed by sonication. The soluble fraction was bound to Ni^2+^-agarose resin, washed several times to remove weakly bound proteins, and eluted from the Ni^2+^-agarose resin in a high concentration of imidazole. After dialysis to remove the imidazole, the proteins were cleaved overnight with thrombin. Uncleaved protein and remaining His-tag were removed by using small amounts of Ni^2+^-resin before further purification by gel-filtration chromatography using a Pharmacia Biotech Superdex 75 column. When not used immediately, proteins were flash-frozen and stored at − 80 °C.

### Equilibrium measurements

The stability of the CAfn2 wild-type and mutant proteins was determined by equilibrium urea denaturation in 50 mM sodium acetate buffer, pH 5.0 (15 mM HOAc, 35 mM NaOAc) at 25 °C. The solutions were left to equilibrate at 25 °C for at least 2 h before measurements were recorded. All experiments were carried out in thermostatted cuvettes at 25 °C. The experiments used an excitation wavelength of 280 nm, and an emission wavelength of 360 nm. Data were fit to an equation describing a two-state transition.^43^

### Change of free energy on mutation

The change of free energy on mutation, ΔΔ*G*_D–N_, was determined using equation (1):^54^

(1) $$\Delta \Delta G_{D-N}=\langle m\rangle \left({\left[urea\right]}_{50\%}^{wt}-{\left[urea\right]}_{50\%}^{mut}\right)$$

Where [urea]_50%_ is the concentration of urea at which 50% of the protein is unfolded for wild-type (wt) and mutant (mut) proteins, and <*m*> is the mean *m*-value determined from all measurements on wild-type and mutant proteins.

### Kinetic measurements

All kinetic experiments were done using an Applied Photophysics stopped-flow fluorimeter. The excitation wavelength was 280 nm and the emission was monitored at wavelengths > 320 nm. All experiments were carried out in 50 mM sodium acetate buffer (pH 5.0) at 25 °C. The final concentration of all proteins was 1 μM. Refolding rates at 0 M denaturant were determined using CAfn2 unfolded at pH 12.4 as described.^18^ Between three and five traces were averaged for each concentration of denaturant. The refolding data were fit to an equation using a single-exponential term. An average refolding *m*-value of 1.06 M^− 1^ was used for mutations L22A, Y36L and L58A. Fitting data to an equation with two exponentials did not improve the residuals. The unfolding data were fit to an equation describing a single-exponential process with curvature.

### Φ-Value analysis

The Φ-value for folding was determined using equation (2):^54^.

(2) $$\Phi =\frac{\Delta \Delta G_{D-‡}}{\Delta \Delta G_{D-N}}$$

where ΔΔ*G*_D–‡_ is the change in the difference in free energy between D and the transition state (‡) upon mutation and calculated from refolding data as follows:

(3) $$\Delta \Delta G_{D-‡}=RT\ln(k_{f}/k_{f\prime })$$

where *k*_f_ and *k*_f′_ are refolding rate constants for wild-type and mutant proteins (at 0 M urea), respectively.

## Figures and Tables

**Table 1:** Structural details at each position mutated in CAfn2

Mutant	Position^a^	SASA (%)	Deleted contacts: residue number (number of contacts deleted)^b^
L10A	A3	0	11(2), 22(12), 23(1), 24(17), 66(1), 67(2), 68(9), 81(6), 82(1), 84(9)
S12A	A2	11	11(1), 15(1), 20(2), 22(1), 84(3), 86(3)
I20A	B2	0	12(4), 15(10), 21(2), 22(6), 55(6), 58(5), 66(13), 86(7), 88(1)
L22A	B3	0	10(11), 12(3), 20(6), 24(3), 38(6), 53(3), 55(4), 66(13), 68(3), 84(8), 86(2)
V33A	BC-loop	3	4(5), 27(4), 29(1), 30(2), 34(2), 36(6), 70(2), 72(2)
Y36L	C5	4	7(7), 24(20), 26(4), 27(8), 33(5), 51(3), 70(4)
Y36F	C5	4	7(1), 24(3), 26(1), 27(2), 33(2), 51(1), 70(1)
V38A	C4	0	22(7), 24(4), 39(1), 45(1), 46(4), 48(3), 53(2), 55(5), 66(6), 67(1), 68(4)
N40A	C3	18	45(3), 55(1), 58(3), 64(14), 65(2), 66(15)
L44A	C′3	25	37(11), 38(1), 39(15), 45(2), 47(5)
T46A	C′4	10	38(4), 45(1), 47(2), 48(4), 53(1), 54(2), 55(7)
V48A	C′5	8	24(9), 36(3), 38(3), 46(4), 52(1), 53(2), 54(2), 68(1)
A53G	E4	0	22(4), 24(4), 38(3), 46(2), 48(3), 52(2), 54(2), 55(2), 68(1)
I55A	E3	3	20(7), 22(5), 38(6), 40(2), 45(2), 46(8), 53(2), 54(1), 56(1), 58(5), 66(20)
L58A	E2	2	20(7), 40(3), 55(7), 64(23), 66(10), 88(7)
Y64L	F2	11	40(4), 58(15), 59(4), 62(12), 66(1), 88(9)
Y64F	F2	11	58(3), 59(1), 62(3), 88(2)
F66L	F3	0	20(9), 22(2), 38(2), 40(10), 45(1), 55(12), 58(10), 64(12), 86(4), 88(1)
V68A	F4	0	7(5), 10(7), 22(2), 24(30), 36(5), 38(4), 48(1), 53(1), 67(1), 81(4)
A70G	F5	0	7(3), 27(2), 33(3), 36(8), 4(3), 69(1), 71(1), 78(2)
S81A	G4	0	7(1), 10(4), 67(3), 68(3), 80(1), 82(1)
V84A	G3	7	10(8), 11(1), 12(4), 22(5), 66(3), 82(1), 86(4)
V86A	G2	6	12(4), 15(6), 20(6), 22(2), 64(3), 66(8), 84(5), 88(1)

a Each residue is described by strand and core layer (see Figure 3).

b Side-chain — side chain contacts within 6 Å deleted on mutation.

**Table 2:** Changes in stability and refolding kinetics for mutants of CAfn2

Protein	Position	*m*_D–N_ (kcal mol^− 1^ M^− 1^)	[urea]_50%_ (M)	ΔΔ*G*_D–N_ (kcal mol^− 1^)	*k*_f_ at 0 M urea (s^− 1^)	*m*_kf_ (M^− 1^)	Φ at 0 M urea
Wild-type		1.08 ± 0.14	6.20 ± 0.14		4.23 ± 0.11	0.89 ± 0.01	
L10A	A3	1.19 ± 0.05	4.01 ± 0.03	2.52 ± 0.17	4.70 ± 0.04	1.00 ± 0.04	0.03 ± 0.01
S12A	A2	1.00 ± 0.03	6.29 ± 0.05	−0.10 ± 0.18			ND
I20A	B2	1.15 ± 0.05	3.50 ± 0.03	3.11 ± 0.18	1.35 ± 0.10	1.12 ± 0.07	0.22 ± 0.02
L22A	B3	1.16 ± 0.11	1.39 ± 0.12	5.54 ± 0.25	0.44 ± 0.03	a	0.24 ± 0.01
V33A	BC-loop	1.16 ± 0.07	4.66 ± 0.04	1.77 ± 0.18	3.12 ± 0.22	0.88 ± 0.03	0.10 ± 0.03
Y36F	C5	1.25 ± 0.12	4.66 ± 0.06	1.77 ± 0.18	4.24 ± 0.18	1.04 ± 0.03	0.00 ± 0.02
Y36L	C5	1.46 ± 0.34	1.02 ± 0.10	5.97 ± 0.24	0.92 ± 0.10	a	0.15 ± 0.01
F36L	C5	1.46 ± 0.34	1.02 ± 0.10	4.20 ± 0.10	0.92 ± 0.10	a	0.22 ± 0.02
V38A	C4	1.27 ± 0.06	2.56 ± 0.03	4.19 ± 0.19	0.25 ± 0.02	1.46 ± 0.05	0.40 ± 0.02
N40A	C3	0.99 ± 0.08	5.33 ± 0.10	1.00 ± 0.20	4.29 ± 0.19	0.87 ± 0.82	0.01 ± 0.03
L44A	C′3	1.25 ± 0.05	3.79 ± 0.03	2.78 ± 0.18	0.61 ± 0.03	1.17 ± 0.03	0.40 ± 0.03
T46A	C′4	0.89 ± 0.03	7.49 ± 0.04	− 1.49 ± 0.17	3.08 ± 0.52	0.88 ± 0.02	0.36 ± 0.05
V48A	C′5	1.10 ± 0.06	4.83 ± 0.04	1.58 ± 0.17	1.28 ± 0.07	1.08 ± 0.02	0.45 ± 0.05
A53G	E4	1.18 ± 0.03	4.18 ± 0.02	2.33 ± 0.17	2.48 ± 0.16	1.23 ± 0.04	0.14 ± 0.02
I55A	E3	1.13 ± 0.08	3.05 ± 0.05	3.63 ± 0.19	0.37 ± 0.05	1.08 ± 0.07	0.40 ± 0.03
L58A	E2	1.13 ± 0.10	1.63 ± 0.05	5.26 ± 0.21	1.06 ± 0.06	a	0.16 ± 0.01
Y64L	F2	1.26 ± 0.07	2.40 ± 0.03	4.38 ± 0.19	3.09 ± 0.14	1.14 ± 0.04	0.04 ± 0.01
Y64F	F2	1.31 ± 0.07	4.88 ± 0.03	1.52 ± 0.17	3.82 ± 0.17	0.94 ± 0.02	0.04 ± 0.02
F64L	F2	1.26 ± 0.07	2.40 ± 0.03	2.86 ± 0.12	3.09 ± 0.14	1.14 ± 0.04	0.12 ± 0.03
F66L	F3	1.08 ± 0.06	4.03 ± 0.05	2.50 ± 0.18	3.13 ± 0.08	1.01 ± 0.02	0.07 ± 0.01
V68A	F4	1.19 ± 0.07	2.67 ± 0.05	4.07 ± 0.20	0.74 ± 0.04	1.36 ± 0.04	0.25 ± 0.01
A70G	F5	1.22 ± 0.03	4.57 ± 0.02	1.88 ± 0.17	1.77 ± 0.06	1.04 ± 0.02	0.27 ± 0.03
S81A	G4	1.13 ± 0.09	4.97 ± 0.06	1.42 ± 0.18	4.84 ± 0.22	0.92 ± 0.02	−0.06 ± 0.02
V84A	G3	1.22 ± 0.06	4.76 ± 0.03	1.66 ± 0.17	3.66 ± 0.17	0.94 ± 0.02	0.05 ± 0.02
V86A	G2	1.15 ± 0.05	4.95 ± 0.15	1.44 ± 0.24	4.19 ± 0.14	0.96 ± 0.01	0.00 ± 0.02

ND Not done: No Φ-value was determined for S12A because ΔΔ*G*_D–N_ ∼ 0. Previous studies have indicated that where ΔΔ*G*_D–N_ > 0.6 kcal mol^− 1^, the corresponding Φ-value can be considered reliable.^58^ The errors reported for *m*_D–N_ and *k*_f_ are standard errors from the fits of the data. Errors in ΔΔ*G*_D–N_ and Φ were determined by standard error propagation methods. ^a^Where the mutation induced a large change in stability there were too few points to fit the refolding *m*-value with accuracy. These mutant chevrons were fit with a fixed average *m-*value of 1.06 M^− 1^.
