# Supplementary material for: Plasticity Within the Obligatory Folding Nucleus of an Immunoglobulin-like Domain
Source: J Mol Biol. 2008 Jan 11;375(2):547–59. doi: 10.1016/j.jmb.2007.09.088 (PMC2291451; doi:10.1016/j.jmb.2007.09.088)
Supplement: Supplementary Figure 1 [file applic1.pdf]

a

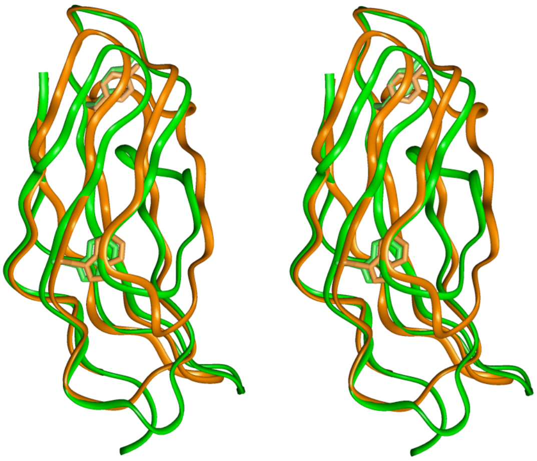

b

```
                  βA            βB               βC
CAfn2 hmaPTAPTNLASTAQTtsSITLSWTASTDnvgvtGYDVYNg........   41
TNfn3 ..rLDAPSQIEVKDVTdtTALITWFKPLAe..idGIELTYgikdvpg..   42
```

```
           βC'       βE              βF                 βG
Query taLATTVT..GTTATISGLAADTSYTFTVKAKdaaGNVSAasnAVSVKT   88
Sbjct drTTIDLTedENQYSIGNLKPDTEYEVSLISRrg.DMSSN.paKETFTT   89
```

Supplementary Figure 1.

Structural alignment of CAfn2 and TNfn3. (a) A stereo view showing an overlay of the backbone traces of CAfn2 (green) and TNfn3 (orange). The front sheet is formed of the A, B and E strands, and the back sheet of the G, F, C and C' strands. The strands are listed from left to right. The two highly conserved residues, tryptophan in the B-strand, and the tyrosine in the EF-loop, are shown in both structures. (b) Structure-based sequence alignment of the CAfn2 and TNfn3. Residues used to overlay the two structures are shown in capital letters. The strands are coloured in blue and the residues forming the putative folding nucleus in TNfn3 are shown in red (Hamill et al. (2000) *J.Mol.Biol.* **297**,165-178).
